# Supplementary material for: Topic modeling for multi-omic integration in the human gut microbiome and implications for Autism
Source: Sci Rep. 2023 Jul 13;13:11353. doi: 10.1038/s41598-023-38228-0 (PMC10345091; doi:10.1038/s41598-023-38228-0)
Supplement: Supplementary file 1 — Supplementary Figures. [file 41598_2023_38228_MOESM1_ESM.pdf]

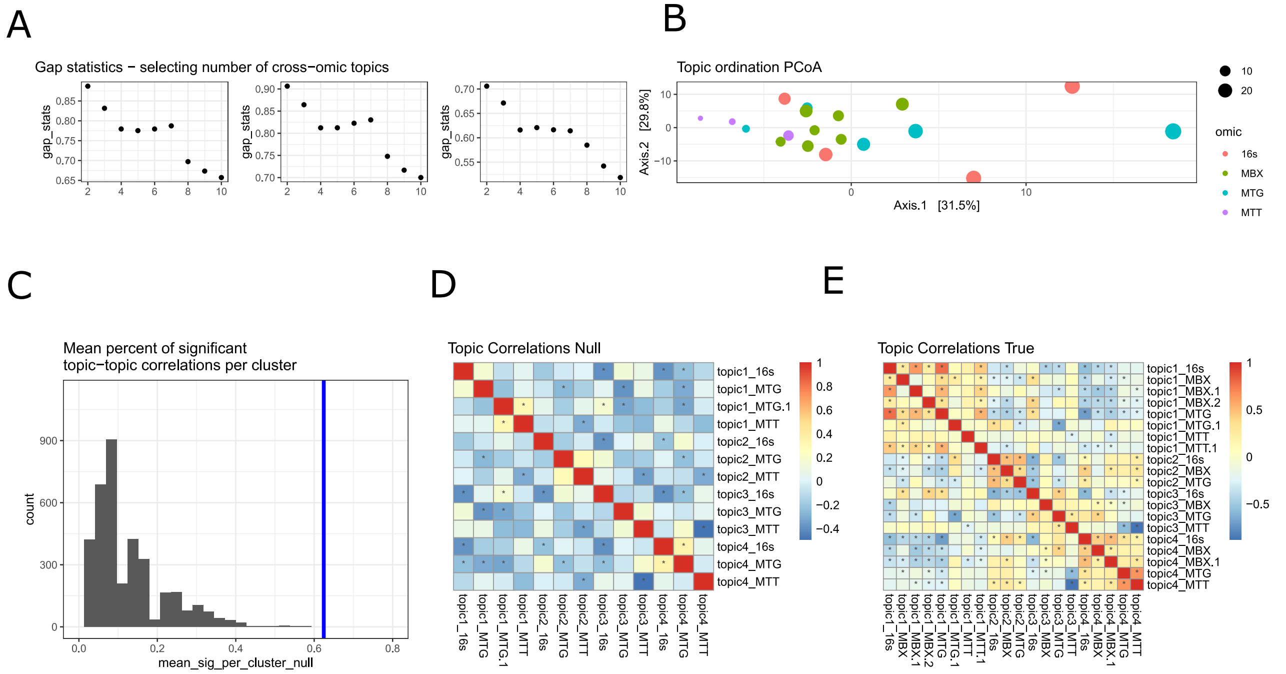

Supplementary Figure 1 Cross-omic topic evaluation

Gap statistics (within/between cluster distances) were calculated using manhattan, euclidean, and inverse correlation distance metrics for 2-10 cross-omic topics. The elbow method was used to select 4 cross-omic topics (A). Topics were visualized by PCoA on the topic distribution across samples using manhattan distance. Size of points represents the total weight attributed to a topic across all samples (how common the topic is) and color represents omic of origin (B). Topic-topic correlations within a cross-omic topic cluster are significantly more correlated than would be expected by random chance. The mean number of significant (spearman correlation $p<0.05$) topic-topic correlations within a cluster using null simulated data (15 permutations) is between 0 and 0.4. The true number mean of significant correlations is over 0.6 (C). Topic-Topic correlation matrix using null simulated data (D). Topic-Topic correlation matrix using true data (E). Cells are colored by spearman correlation coefficient and star indicates significant ($p<0.05$). Multiple topics from the same omic dataset may be in the same cross-omic topic, denoted by ".1" or ".2", etc.

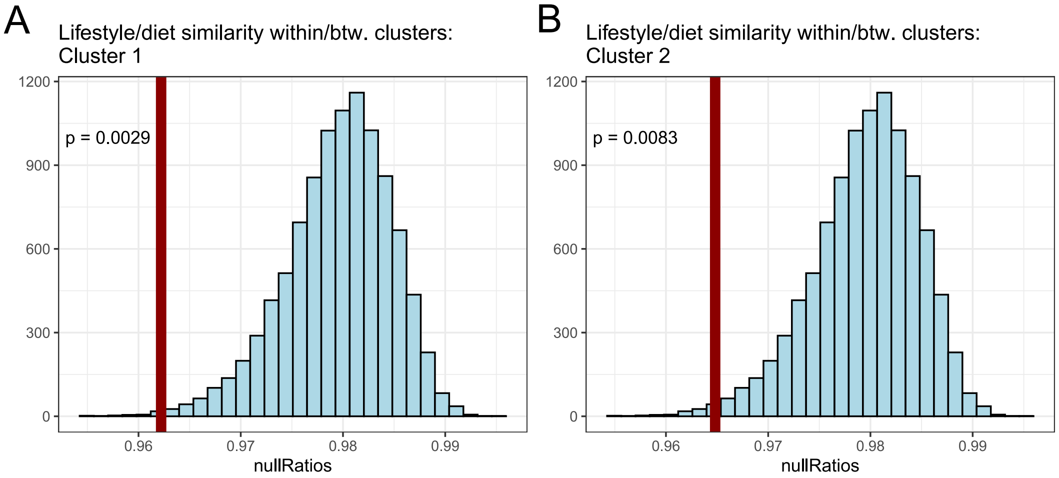

Supplementary Figure 2 Samples in the same topic-derived clusters shared similar dietary patterns. Average within/between cosine distances based on dietary information among samples that belong to cluster 1 (A) and cluster 2 (B) are shown by the red line. Histogram distributions are formed by randomly assigning cluster membership while maintaining cluster size, and re-calculating diet-based cosine distances 10,000 times. P-values are calculated as the percent of null ratio values that are less than the true ratio value.

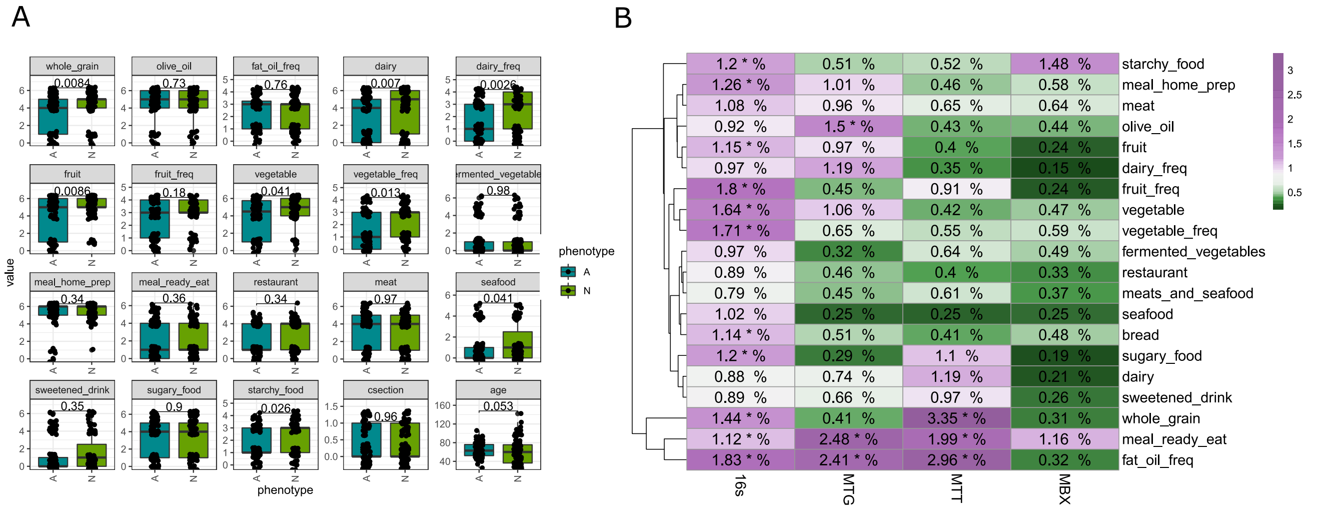

Supplementary Figure 3 Dietary and lifestyle characteristics between autistic and typically developing children compared using a Wilcox rank sum test (A). Association between individual dietary variables and omic data (B). Omic data was normalized using relative log expression (RLE), numbers in the cell are variance explained or R^2 values (as percents) of a permanova test, and a star indicates a significant association by permanova test

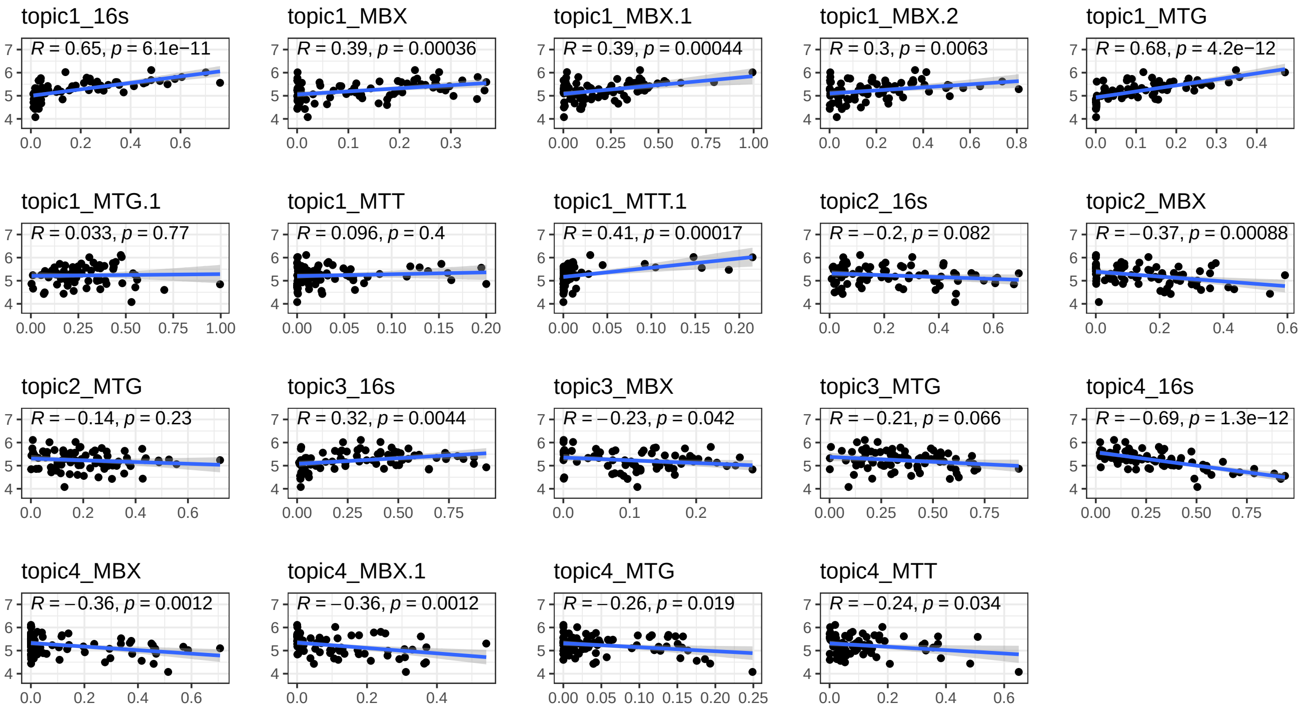

Supplementary Figure 4 Correlation between topic values and 16S Shannon diversity. Lines and confidence intervals are linear regression fits, while R and p values are spearman correlations.

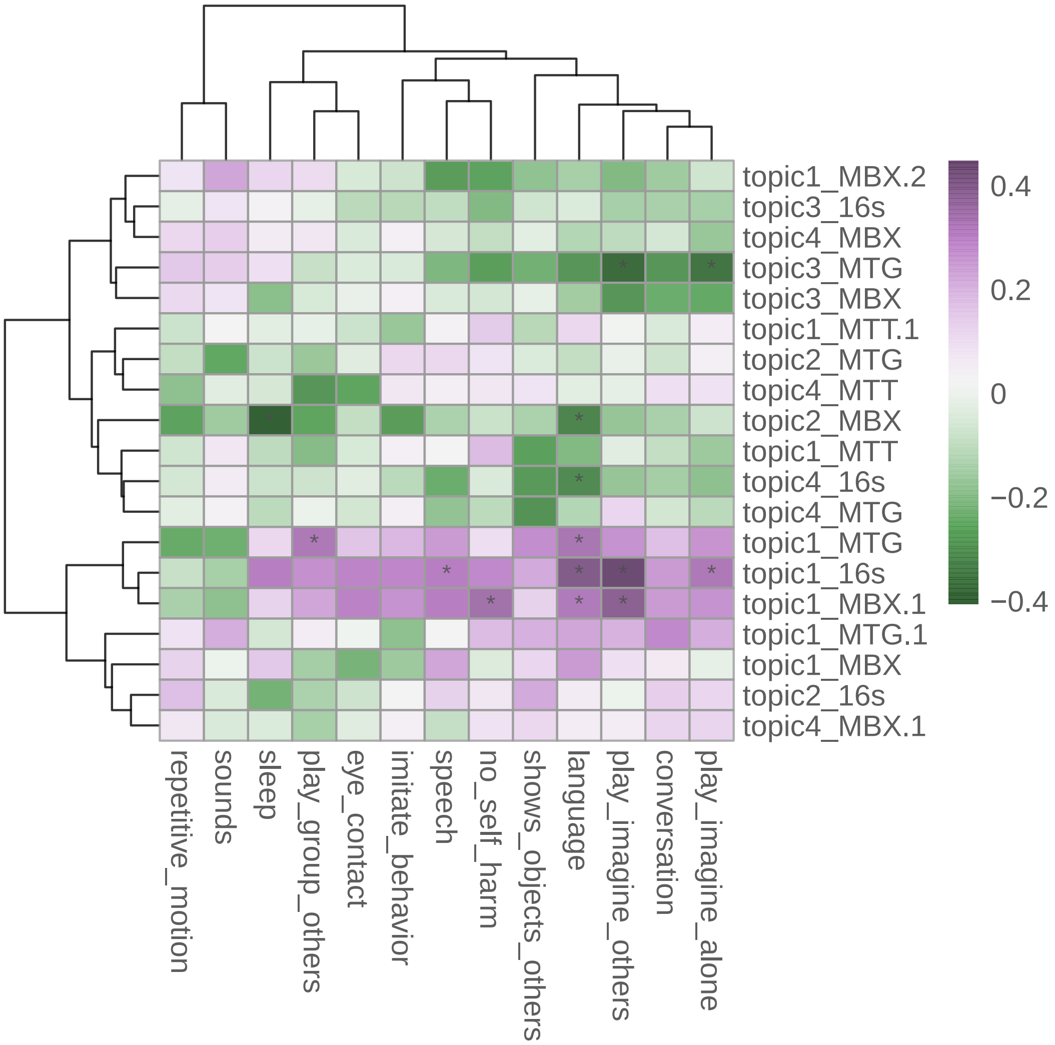

Supplementary Figure 5 Correlations between topics and specific Autism-related behaviors, as determined by parent-completed questionnaire. Questionnaire was only completed for autistic children. Cells are colored by the spearman correlation test ρ value, and a star indicates significant ($p<0.05$).

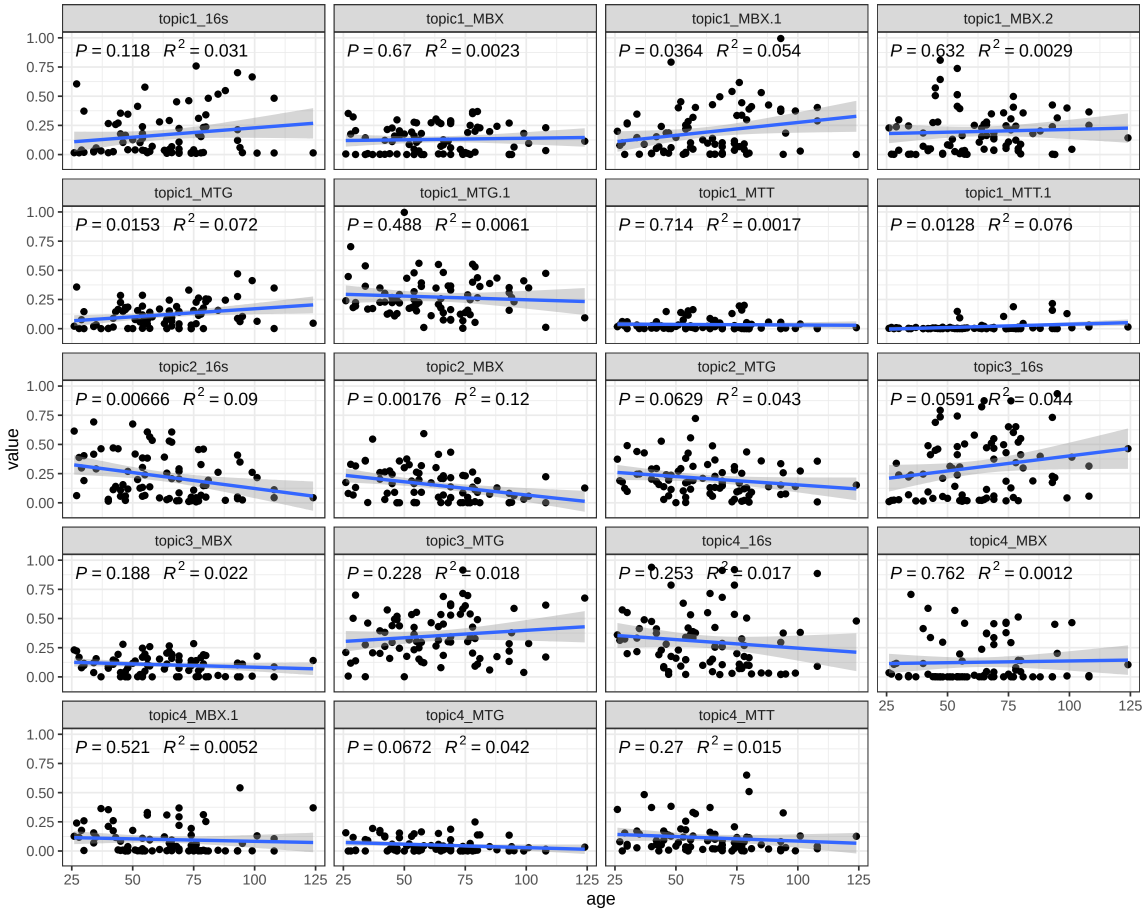

Supplementary Figure 6 Spearman correlation between age (in months) and topic values. Topic 2 most consistently correlates inversely with age, and Topic 1 correlates with age in some omics.

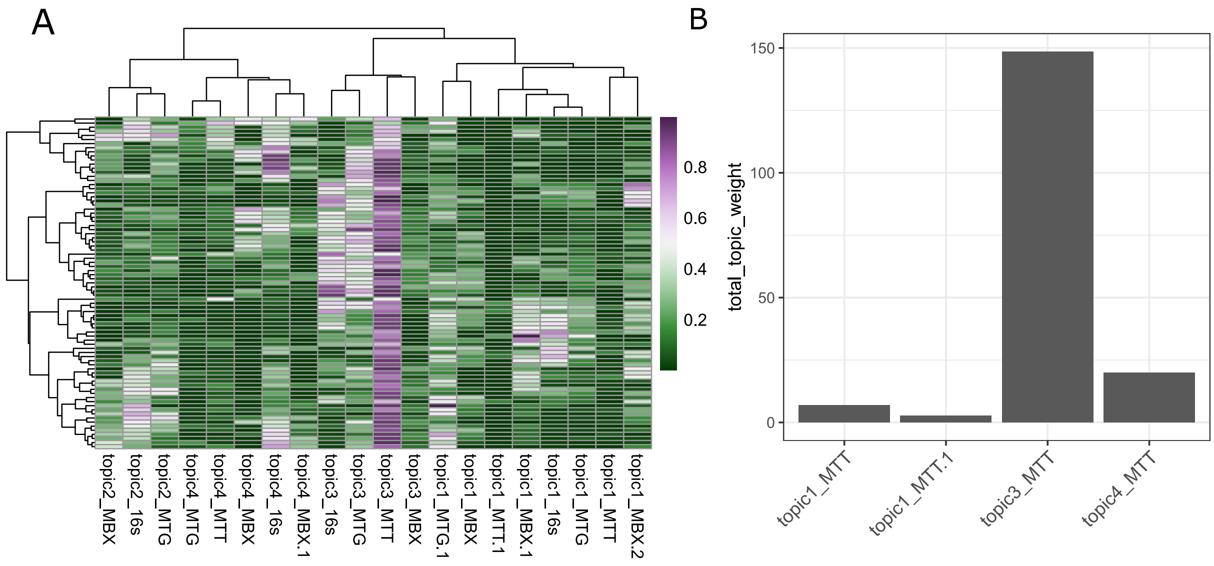

Supplementary Figure 7 MTT topic 3 was removed due to failure to low variability across samples and excessively high topic attribution across samples. MTT topic 3 has high topic values across nearly all samples, and far exceeds all other topic value magnitudes (A). MTT topic 3 has orders of magnitude higher topic attribution across samples compared with other MTT topics (B).

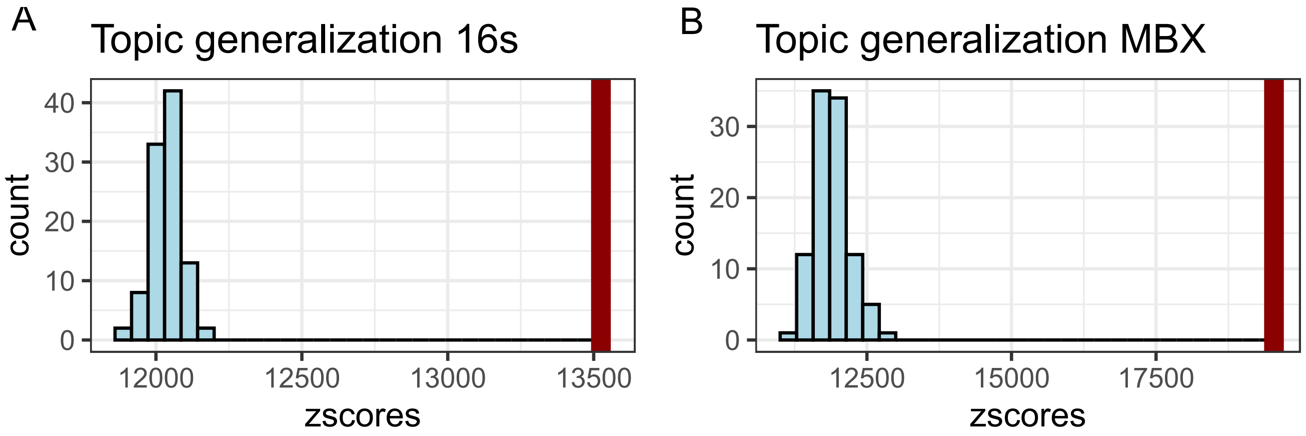

Supplementary Figure 8 Topics define consistent feature-feature relationships regardless of dataset. Feature-feature cosine distance matrices were calculated on the basis of feature distribution across topics per dataset. Then, a mantel-based permutation test was used to compare feature-feature distance matrices between David et. al 16s data and 16s data from this study (A). and between Telleria et. al metabolomics data and data from this study (B).

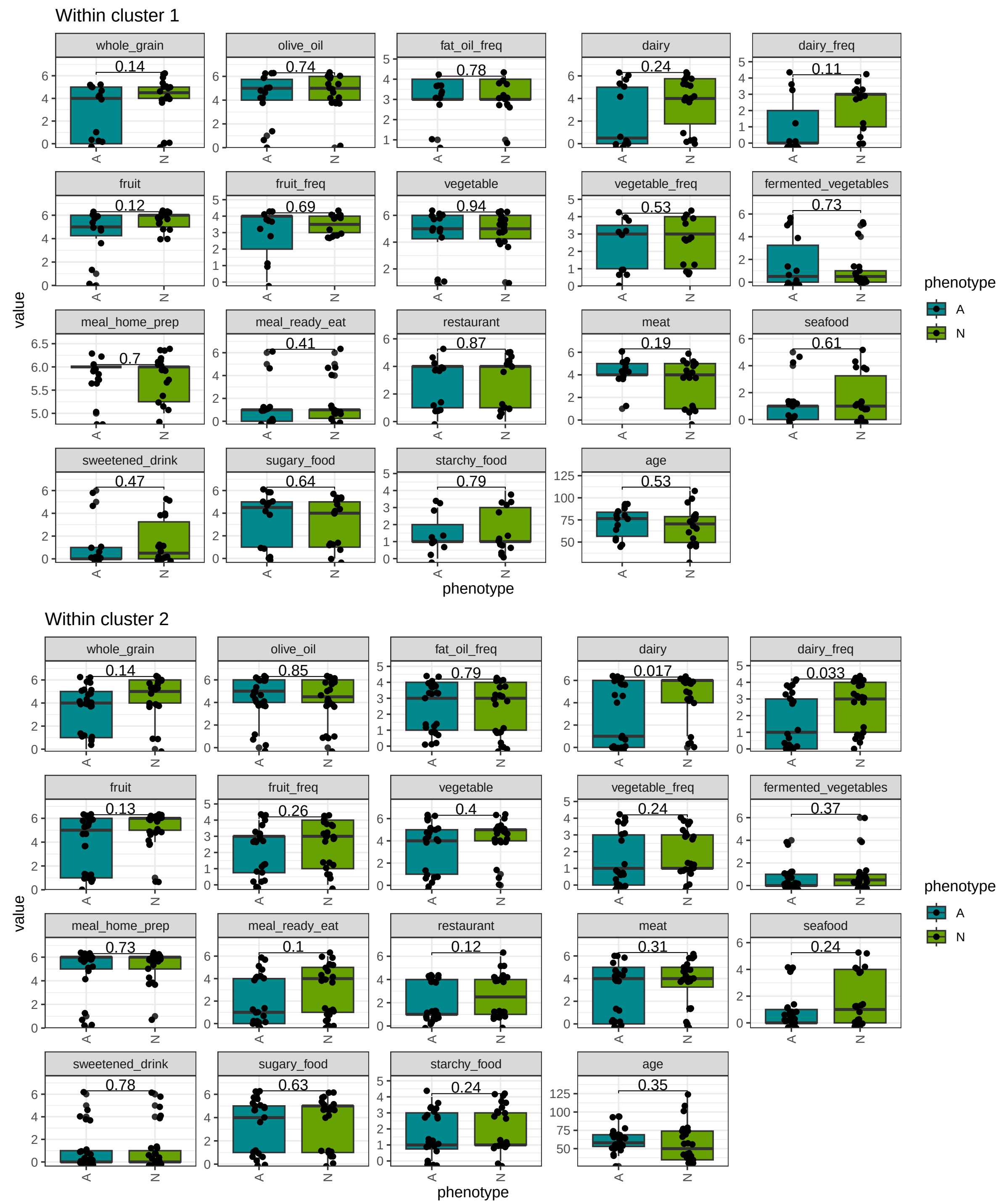

Supplementary Figure 9 Wilcox rank sum test on dietary variable differences between Autistic and typically developing participants per sample cluster.

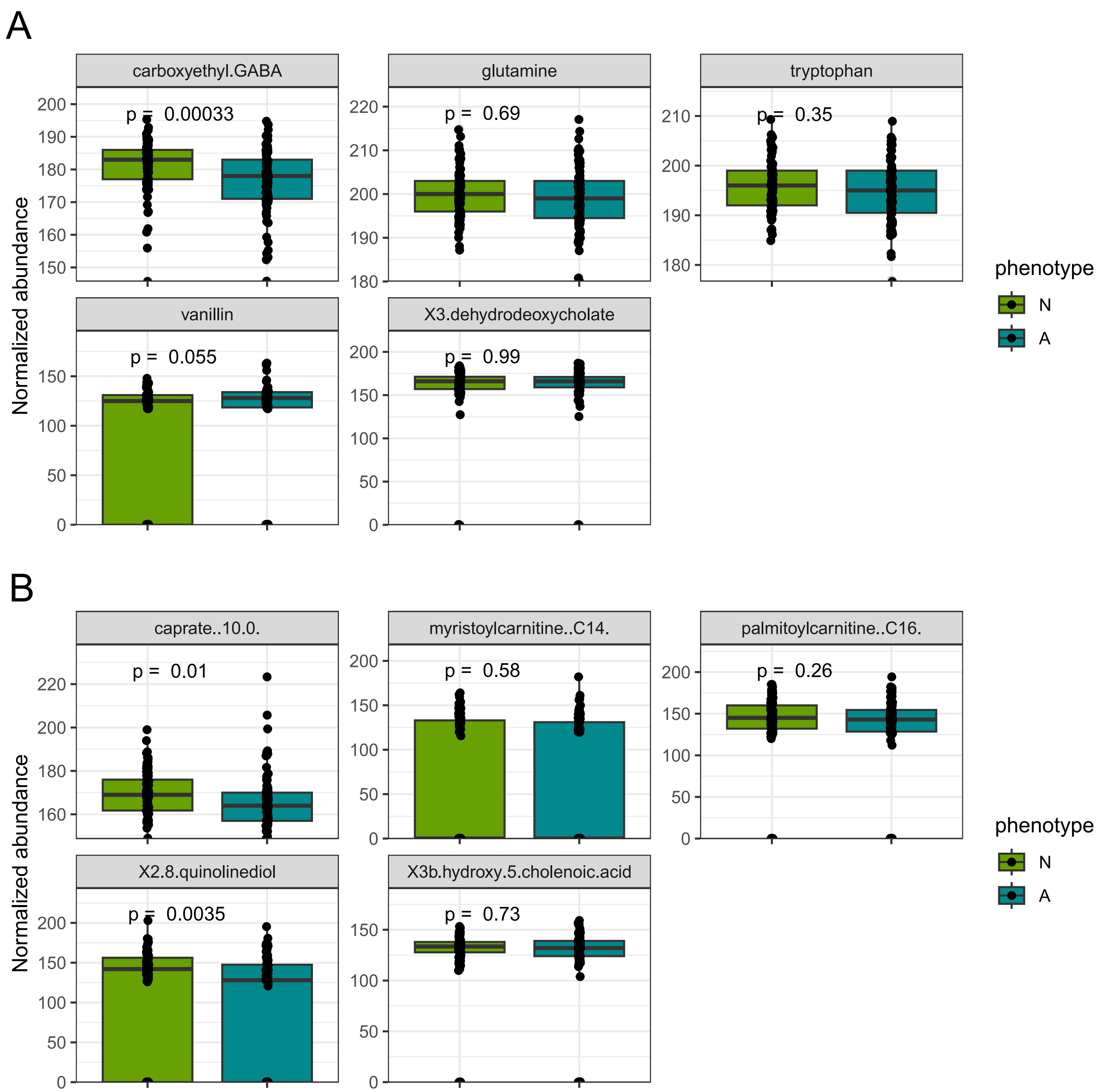

Supplementary Figure 10 Differential analysis of metabolites identified per cluster across the entire cohort. Metabolites identified in cluster 1 seen in (A) and metabolites identified in cluster 2 seen in (B). P values are reported using Wilcox rank sum test.

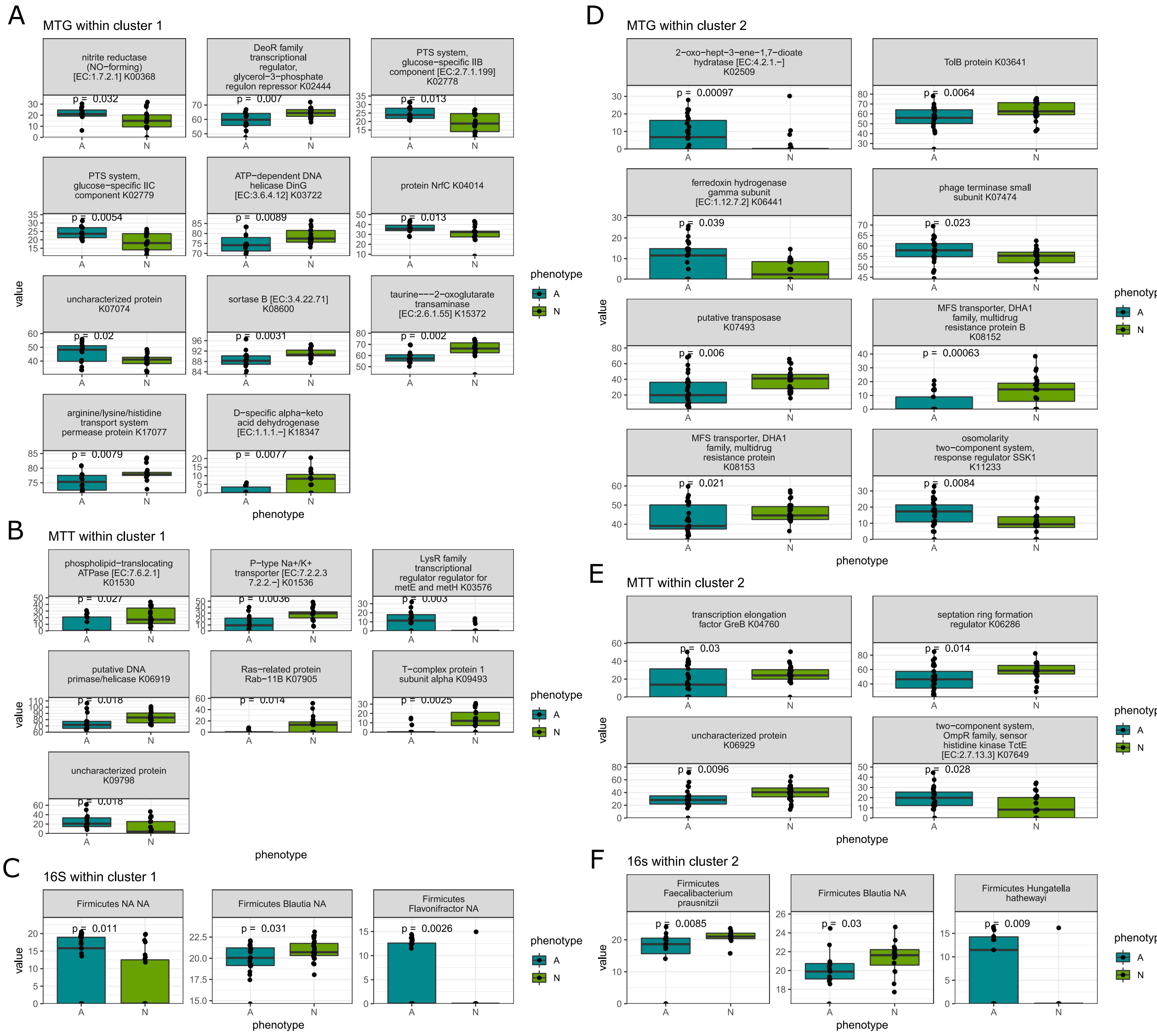

Supplementary Figure 11 Differential analysis of MTG, MTT, and 16S features between autistic and typically developing children within sample clusters as determined by topics. Within cluster 1, MTG features (A), MTT features (B), 16S features (C). Within cluster 2, MTG features (D), MTT features (E), 16s features (F). Features are first filtered using the Boruta package, and then p values are reported using Wilcox rank sum test.

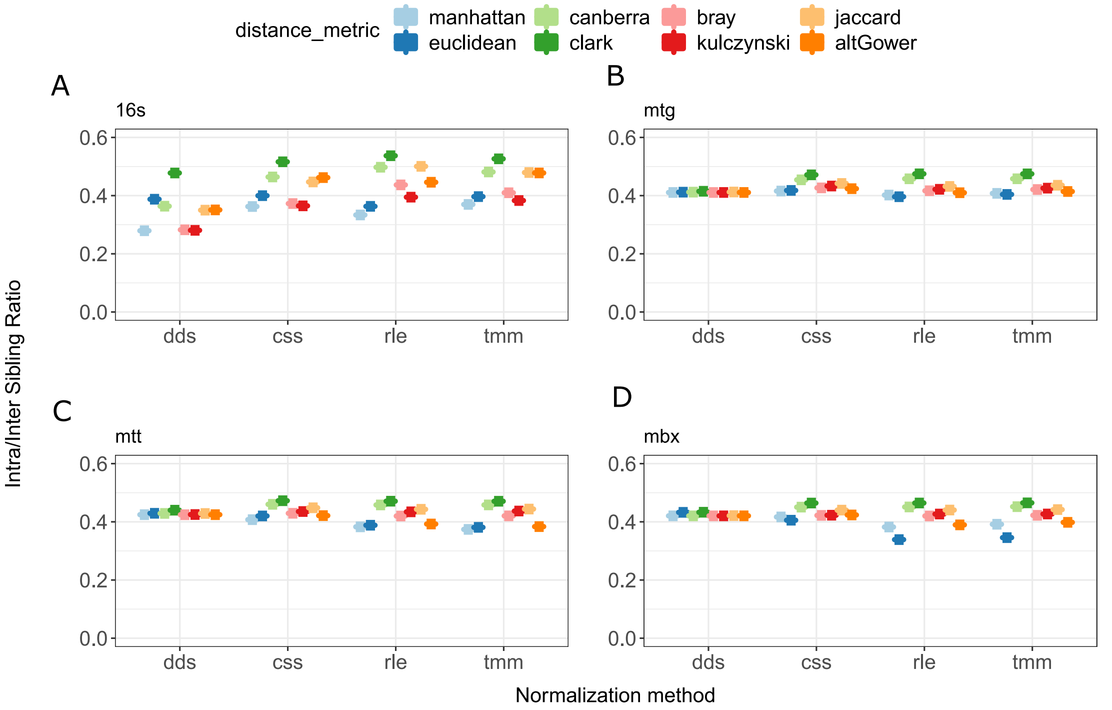

Supplementary Figure 12 Choosing a normalization method per omic datasets. Normalization methods that minimized the distance between siblings vs. the distance between all unrelated samples there selected. When differences in normalization performance were negligible, the majority method was used. 16S DeSeq2 (A), MTG RLE (B), MTT RLE (C), MBX RLE (D)

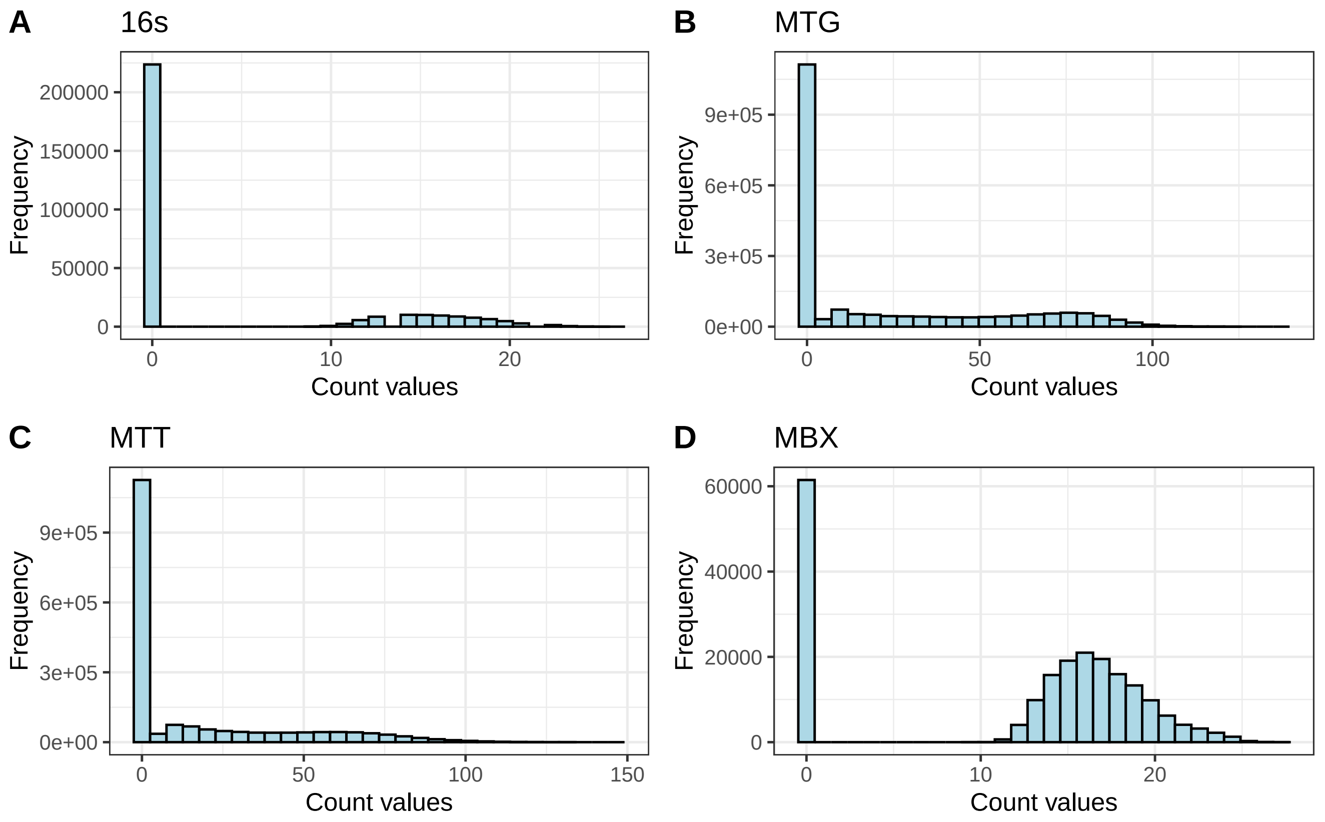

Supplementary Figure 13 Distribution of feature values across omic datasets. Histogram of feature counts across 16S (A), MTG (B), MTT(C), MBX (D) after normalization. 16s/MBX and MTG/MTT data distributions are most similar.

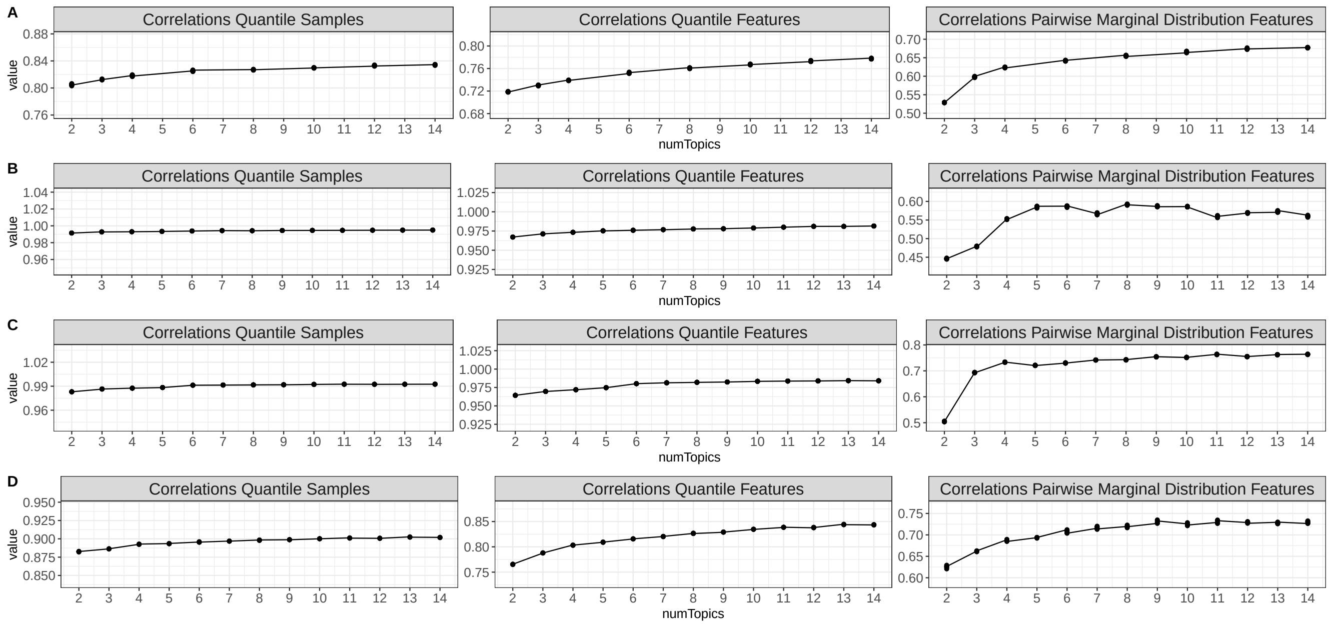

Supplementary Figure 14 Selecting number of topics per model. To evaluate model fit, sample data was simulated using each model, and simulated and true data were compared using three metrics: correlation of sample quantiles, correlation of feature quantiles, and marginal pairwise distribution of features. Number of topics was selected using the elbow method. The final number of topics selected were 4 for 16s data (A), 5 for MTG data (B), 4 for MTT data (C), and 7 for MBX data (D).
